# A novel brain targeted plasma exosomes enhance the neuroprotective efficacy of edaravone in ischemic stroke

**DOI:** 10.1049/nbt2.12003

**Published:** 2021-02-02

**Authors:** Lin Guo, Junlu Pan, Fang Li, Liang Zhao, Yijie Shi

**Affiliations:** ^1^ School of Pharmacy Jinzhou Medical University Jinzhou People's Republic of China

## Abstract

Ischemic stroke is often involved in the excessive production of reactive oxygen species (ROS), which aggravate ischemic injury. Edaravone (EDV) as an efficient free radical scavenger has demonstrated the effective neuroprotective effects in the therapy of ischemic stroke. Although EDV promotes ischemic recovery by inhibiting the generation of ROS, its poor safety and bioavailability limit its clinical applications. Herein, we developed plasma exosomes (EXO) containing EDV (EXO + EDV) for improving short‐term functional and histological outcomes for stroke treatment. The results showed that EXO + EDV improved brain targeting based on the transferrin–transferrin receptor interaction, and the safety and bioavailability of EDV were also significantly increased. Furthermore, compared with EDV, EXO + EDV significantly rescued ischemic damage in brain tissue by reducing infarct area and improving neurological performance in the acute stage of stroke (first 7 days).

## 1 INTRODUCTION

Ischemic stroke is the second leading cause of death and accounts for 9% of deaths worldwide. Reactive oxygen species (ROS) have been implicated in ischemic stroke induced brain injury [1]. After ischemic stroke occurs, a large amount of ROS are produced in the brain tissue. ROS leads to the death of brain endothelial cells and neurons by oxidising and destroying brain microvascular endothelial cell membranes, as well as neurons, thus aggravating severe brain damage and cerebral infarction [2,3]. Edaravone (EDV, 3‐methyl‐1‐phenyl‐2‐pyrazolin‐5‐one) as a strong free radical scavenger has been clinically applied for the treatment of ischemic stroke through eliminating oxygen free radicals, inhibiting cell peroxidation and reducing tissue damage [4,5]. However, only available commercial supplied EDV formulation for the treatment of acute ischemic stroke is its intravenous injections [6,7], which has showed some pitiable shortcomings in that it often induces strong hemolysis effect duo to the use of propylene glycol (PG) as a co‐solvent [8]. Owing to EDV's short half‐life and low bioavailability, a relatively high dose (30 mg twice/day/person) of EDV has to be injected, which results in complications such as renal function disorder. In addition, as the stability of EDV is poor, it needs to be temporarily prepared and completely administered intravenously within half an hour [9]. All of the above problems affect the effective use of EDV in the clinical treatment of ischemic stroke. Therefore, finding a new strategy to improve safety and bioavailability of EDV for better treatment of ischemic stroke is of great significance.

**Scheme 1:**
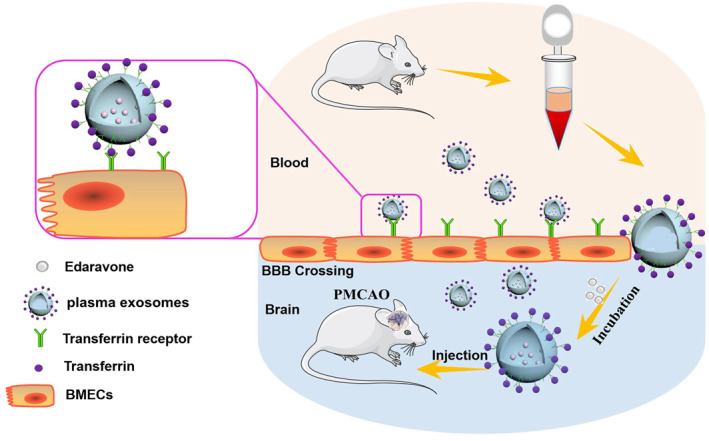
The primary hypothesis of this study. EXO was collected by ultracentrifugation, and EDV was loaded into EXO by co‐incubation for improving safety and bioavailability of EDV. Furthermore, EXO + EDV effectively penetrated through BBB and targeted brain via transferrin–TfR interaction, thereby exerting the attenuation of ischemic injury in PMCAO model rats

The exosomes, characterised by nanovesicles ranging at 40–200 nm with a lipid‐like bilayer structure, are playing an important role on information exchange and substance transfer between cells by delivering miRNAs, mRNAs and proteins to recipient cells [10]. Especially, in the light of advantages of exosomes on low immunogenicity, high biocompatibility and inherent ability to encapsulate exogenous bioactive molecules, it is also considered to be a desirable naturally equipped nanocarrier for incorporating exogenous drugs via different approaches such as co‐incubation, electroporation and transfection [11, 12, 13]. However, exosomes of natural origin are usually easily taken up by organs of the mononuclear phagocyte system (MPS) and show the poor targeting ability for brain [14,15]. Therefore, they further need chemical surface modification, which is usually complex and highly expensive [16,17]. It is newly found that plasma exosomes (EXO) could target the brain by the interaction between transferrin that was expressed at the surface of EXO and transferrin receptor (TfR) on the surface of brain endothelial cells, thus facilitating transport of EXO across the BBB via TfR‐mediated endocytosis [17]. It is reported that dopamine‐loaded blood exosomes targeted to the brain with the mediation of transferrin–TfR interaction and showed better treatment of Parkinson's disease [18]. Therefore, we hypothesized that EXO could deliver drugs across BBB based on the transferrin–TfR interaction. To overcome the above problems associated with the safety and bioavailability of EDV, we fabricated EDV‐loaded EXO (EXO + EDV) by co‐incubating EDV with EXO. We aimed to investigate whether EXO + EDV improved the neuroprotective effects of EDV after permanent stroke. The results demonstrated that compared with traditional EDV injection containing PG serving as a co‐solvent, hemolysis effect of EXO + EDV was demonstrated to have been significantly reduced. In addition, EXO + EDV also contributed to the higher bioavailability by a substantial increase in plasma area under curve (AUC), an extended half‐life (*t*_1/2_) and a significant decrease in the total body clearance (CL) of EDV. As EXO inherited the transferrin, a protein that interacted with endothelial TfR and mediated the lateral migration and diapedesis of EXO across the BBB, it can effectively penetrate through BBB and transfer more proportions of EDV into brain based on the transferrin–TfR interaction (Scheme 1). Furthermore, EXO + EDV alleviated cerebral ischemia injury in the acute stage of stroke (first 7 days) in permanent middle cerebral artery occlusion (PMCAO) model rats by reducing infarct volume, attenuating neurological severity and rescuing the survival of neuron cells.

## 2 MATERIALS AND METHODS

### 2.1 Cell culture

Human brain microvascular endothelial cells (HBMECs) and Hela cells were purchased from American Type Culture Collection (ATCC) and cultured in Dulbecco's modified Eagle's medium (DMEM, Hyclone, UT, USA) containing 10% fetal bovine serum (FBS; Gibco, CA, USA) and 1% penicillin–streptomycin (PS). All cells were maintained in a humidified cell culture incubator at 37°C with 5% carbon dioxide (CO_2_).

### 2.2 Plasma exosomes isolation and characterisation

In order to obtain EDV‐loaded EXO, the blood sample of normal Sprague‐Dawley (SD) rats was collected in a clean EP tube containing 0.1% sodium heparin, and EXO was isolated by gradient centrifugation at 2000 × *g* for 30 min, 10,000 × *g* for 30 min and 100,000 × *g* for 2 h. Finally, EXO was extensively washed and incubated with EDV saturated solution by mild sonification for 24 h at 4°C [19]. The obtained EXO + EDV was washed three times, and EDV in supernatant was obtained and measured by high performance liquid chromatography (HPLC). Encapsulation efficiency (EE%) and loading rate (LR%) of EDV in EXO were calculated using the equation below [Equations (1) and (2)]:

(1) $$EE\left(\%\right)=\;\frac{\text{Weight}\;\text{of}\;\text{initially}\;\text{added}\;\text{EDV}-\text{Weight}\;\text{of}\;\text{EDV}\;\text{in}\;\text{supernatant}}{\text{Weight}\;\text{of}\;\text{initially}\;\text{added}\;\text{EDV}}\times 100$$

(2) $$LR\left(\%\right)=\;\frac{\text{Weight}\;\text{of}\;\text{initially}\;\text{added}\;\text{EDV}-\text{Weight}\;\text{of}\;\text{EDV}\;\text{in}\;\text{supernatant}}{\text{Weight}\;\text{of}\;\text{EXO}+\text{EDV}}\times 100$$

Finally, the morphology and size of EXO and EXO + EDV were examined by atomic force microscopy (FAM, FM‐Nanoview6800; FSM‐PRECISION, Suzhou, China), and the particle size was detected by dynamic light scattering (Zetasizer Nano ZS; Malvern Instruments, Malvern, UK). Western blotting was used to detect the expression levels of the exosomal marker proteins such as CD63 and Alix.

### 2.3 Model of permanent middle cerebral artery occlusion

Male SD rats weighing 250–300 g were provided by the Animal Center of Jinzhou Medical University and approved by the Animal Protection and Use Committee of Jinzhou Medical University. For establishing model of PMCAO, a surgical suture (6‐0 nylon) was used to block blood supply by inserting into the right internal carotid artery.

### 2.4 Cellular uptake assay of plasma exosomes in vitro

In order to achieve the brain targeting, EXO must first be efficiently internalised by HBMECs as an important component of BBB. Therefore, we first investigated the intracellular internalisation and distribution of EXO. HBMECs (ferritin receptor positive cells) and Hela (ferritin receptor negative cells) were seeded in into confocal dishes for 24 h continuous incubation. Next, 1,1′‐dioctadecyl‐3,3,3′,3′‐tetramethylindocarbocyanine perchlorate (DiI)‐labeled EXO was added into the medium and incubated with cells. At predetermined intervals, the cellular distributions of DiI‐labeled EXO were observed by confocal laser scanning microscopy. Hela cells derived exosomes were isolated from the culture medium of Hela cells and also used as ferritin negative exosomes to observe its uptake in HBMECs and Hela cells.

### 2.5 Pharmacokinetic properties of edaravone and edaravone loaded plasma exosomes in vivo

To test the bioavailability of EDV and EXO + EDV in vivo, SD rats of 250–300 g were selected and housed in the same litter for two weeks. They were randomly divided into the EDV group and the EXO + EDV group and given a dose of EDV at a dose of 10 mg/kg via iv administration. The blood samples of SD rats were periodically collected via eye sinus bleeding and immediately centrifuged to obtain plasma. After the proteins in plasma were sedimented with perchloric acid, EDV in supernatant was obtained and measured by HPLC. According to the concentration change of EDV in the blood over time, blood drug concentration–time curve in EDV group and EXO + EDV group were obtained.

In order to evaluate the distribution and biocompatibility of EDV in different tissues, after 8 h of administration of EDV and EXO + EDV, all animals were euthanised and tissues (heart, liver, spleen, lung, kidney, brain) were rapidly collected to determine EDV concentrations by HPLC and biocompatibility by H&E staining test.

### 2.6 Hemolysis analysis

8 ml blood of rats was centrifuged at 1500 rpm for 10 min, and the supernatant was discarded followed by washing twice with 0.9% saline until the supernatant was not red. EDV, EXO + EDV and the combination of PG and EDV (EDV + PG) with the same concentration of EDV were added into the erythrocyte suspension, respectively, for continuous incubation at 37°C. Saline solution and 0.3% Triton were used as negative and positive control groups [20], respectively. The images of hemolysis of blood treated with different samples were captured respectively and analyzed by observing the colour change.

### 2.7 Distinct distribution of plasma exosomes in the ischemic brain

3,3′‐dioctadecyloxacarbocyanine perchlorate (DiO)‐labeled EXO (3 mg/kg) was intravenously injected into rats via tail vein. After 6 h of administration, the rats were euthanised followed by cardiac perfusion. Finally, the brain was taken out and frozen at ‐80°C in an optimal cutting temperature‐embedded medium (Sakura, Torrance, CA, USA). Frozen brain sections of 20 μm thickness were prepared using a cryostat (CM1950, Leica, Germany) and stained with DAPI at room temperature. The distribution of EXO in the ischemic hemisphere of brain was then imaged under a fluorescence microscope (Axiovert 40 CFL, Zeiss, Germany). To further detect the intracellular distribution of EXO in ischemic region, brain slices were obtained and stained with neuron marker NeuN and endothelia specific indicator CD34 to detect the co‐location of EXO with cells in the ischemic region.

### 2.8 2,3,5‐triphenyltetrazolium chloride staining and neurological evaluation

After SD rats were subjected to PMCAO, 1 ml saline and saline of EXO, EDV (10 mg/kg) and EXO + EDV (10 mg/kg) were administered by injection via tail vein daily for consecutive 7 days. In order to evaluate the neuroprotective effects of EXO + EDV for treating ischemic stroke, TTC (2,3,5‐triphenyltetrazolium chloride) staining method was performed according to the previous protocol [21], and the percent infarct size in each rat was calculated [22]. We also used neurological score (Zea‐Longa's 5‐point) to assess the neurological outcomes of treatment with EXO + EDV.

In order to access EXO + EDV induced protection of neuron, we used Nissl staining to stain Nissl body and determine the integrity of basic neuronal structure. According to the previous reports [23], as frozen section was stained in 0.1% cresyl violet solution followed by the series treatment of differentiation, dehydration, clearing and mounting, the Nissl body was stained purple‐blue.

### 2.9 Statistical analysis

All data are presented as means ± SD. Independent‐sample *t*‐tests were used to compare means between two different groups. One‐way analysis of variance (ANOVA) was used to determine the level of significance with GraphPad Prism software, and *P* < 0.05 was considered to be statistically significant.

## 3 RESULTS

### 3.1 Characterization of edaravone loaded plasma exosomes

**FIGURE 1:**
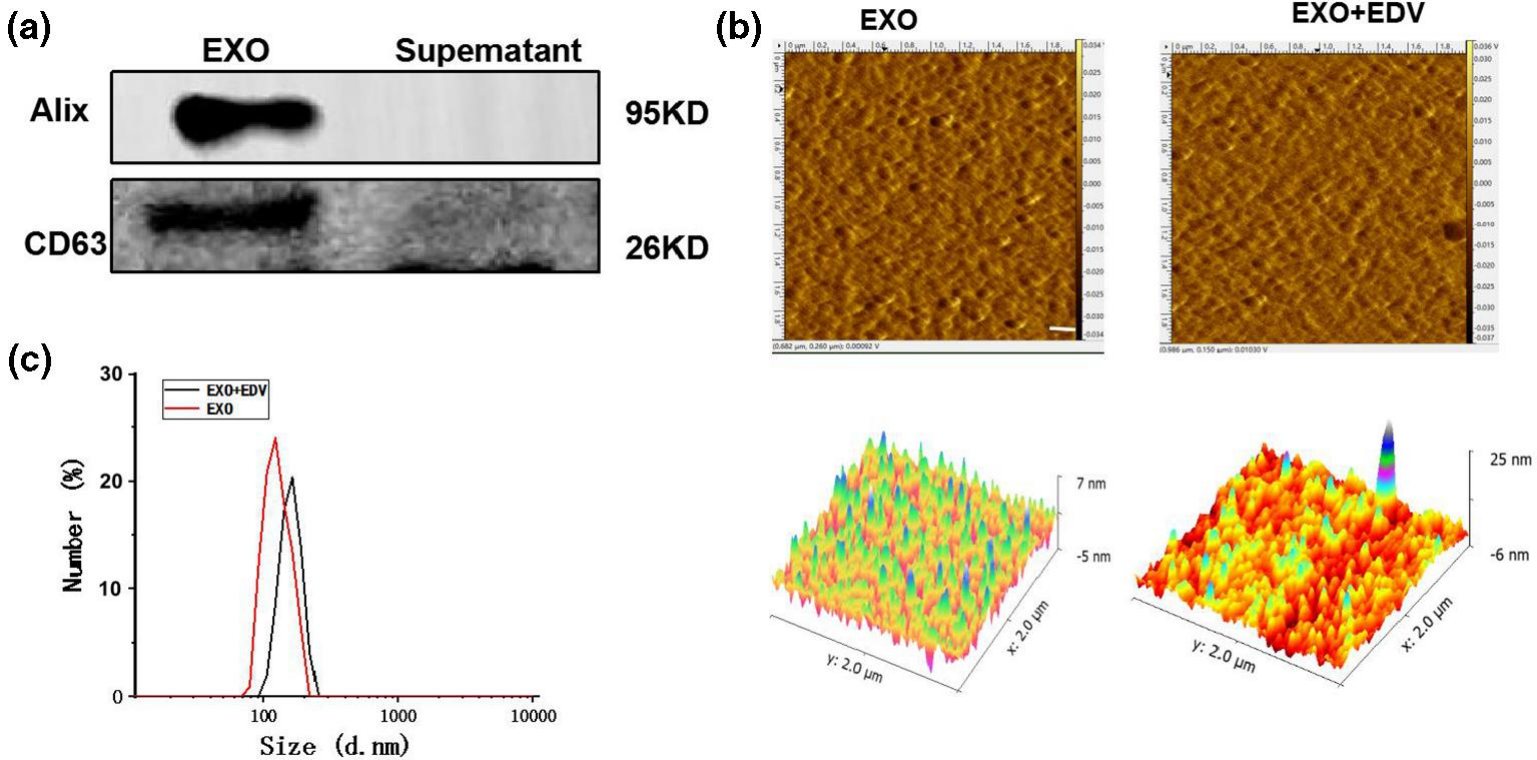
Characterization of EXO and EXO + EDV: (a) Western blotting analysis of the EXO surface markers. (b) Morphology of EXO and EXO + EDV observed by AFM. (c) Particle size distribution of EXO and EXO + EDV measured by DLS

Fresh blood was obtained from normal SD rats, and EXO was isolated from plasma by ultracentrifugation. Western blot analysis (Figure 1a) showed that Alix and CD63 (known exosome markers) were enriched in exosomal particles, and no Alix and CD63 were detected in the supernatant, confirming that EXO had been completely isolated and collected from plasma. According to the results of AFM and DLS (Figure 1b and 1c), EXO and EXO + EDV presented normal morphological characteristics with a diameter of approximately 150  and 165 nm, indicating that the properties of EXO were not affected by the modifications of EDV. EE% and LR% of EDV in EXO were 55.7% and 22.0%.

### 3.2 Pharmacokinetic properties of edaravone and edaravone loaded plasma exosomes in vivo

**FIGURE 2:**
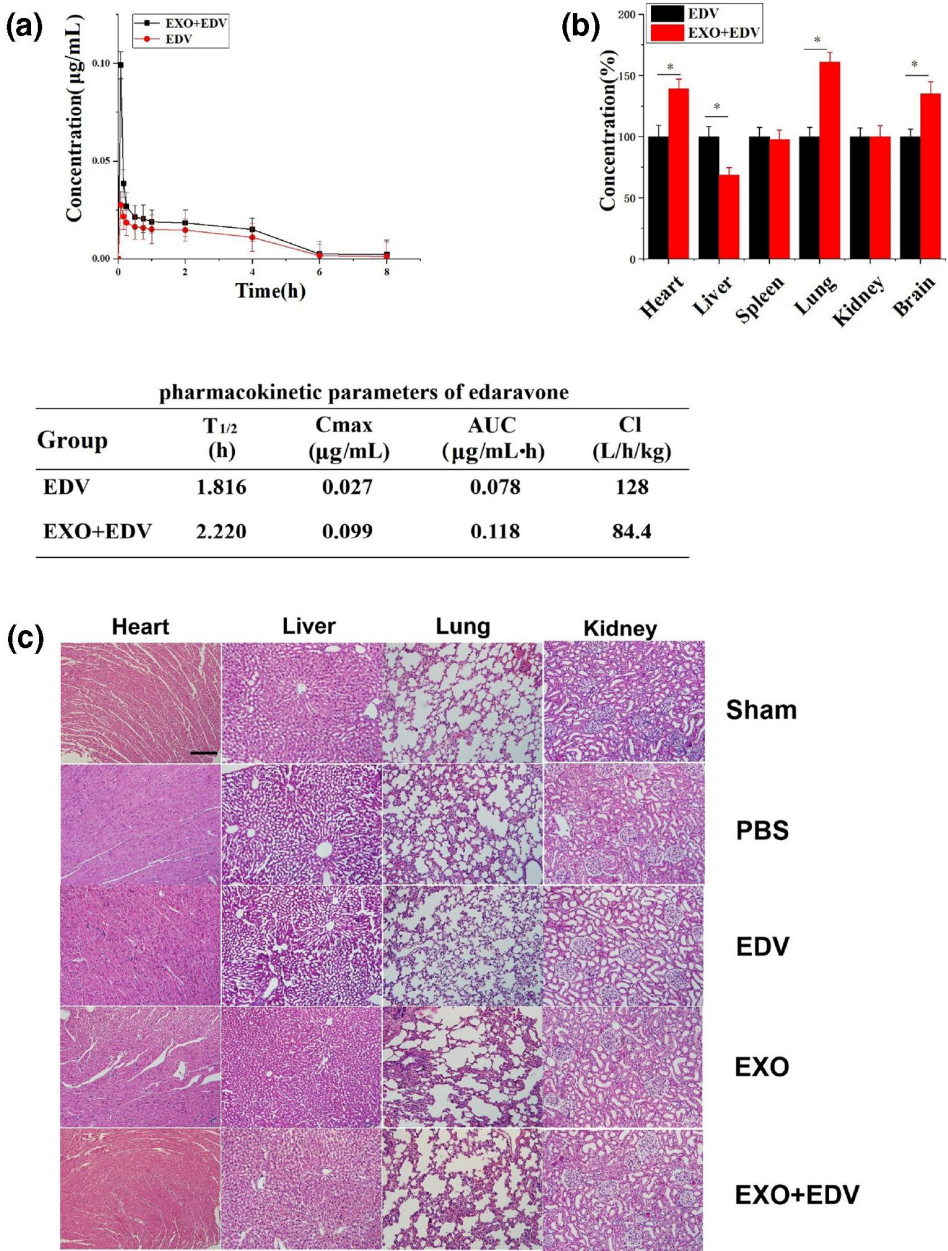
Biodistribution of EXO + EDV and EDV in vivo. (a) The drug concentration–time curve and PK parameters of EDV in rat plasma after single iv administration of EDV and EXO + EDV at the concentration of EDV (10 mg/kg). Data are expressed as means ± SD (*n* = 3). (b) Tissue distributions of EDV in rats treated with EDV and EXO + EDV at 8 h after single iv administration. The concentrations of exosomal EDV in different organs were quantified by comparing with the concentration of EDV in organs in EDV treated group as the control group (100%). Data are expressed as means ± SD (*n* = 3), *P < 0.05. (c) H&E staining of heart, liver, lung and kidney tissue sections in 8 h after iv administering a single dose of different EDV formulations. The scale bar is 200 μm and applies to all figure parts

To assess whether exosomal EDV can increase bioavailability of EDV, EDV and EXO + EDV were administered intravenously (i.v.) in SD rats for evaluating their pharmacokinetic profiles. The pharmacokinetic parameters of EDV in two groups were obtained by software and time‐dependent plasma concentration profiles of EDV in rats were shown in Figure 2a. The results demonstrated that compared with EDV, EXO + EDV showed an extended half‐life (*t*_1/2_) and higher maximum plasma concentration. In addition, AUC was significantly increased, and the total CL of EDV was also decreased in EXO + EDV treated group. Taken together, all these results confirmed that compared with EDV, EXO + EDV contributed to more drug accumulation and slower clearance rate.

In order to detect the distribution of EDV in the body after iv administration, heart, liver spleen, lung, kidney and brain of SD rats at 8 h after administration were collected, and EDV in these organs was extracted after treatment of perchloric acid. The concentration of EDV in organs was detected by HPLC and calculated according to the standard curve. The results (Figure 2b) showed that at 8 h after administration, EXO + EDV injection increased the accumulation of EDV in the brain, heart and lung compared with those following EDV treatment. To be specific, the concentration of EDV in EXO + EDV‐treated group was higher in the brain than that in the EDV‐treated group, indicating that the EXO + EDV could enhance brain‐targeting of EDV. Interestingly, we also found that EXO + EDV injection increased the accumulation of EDV in heart and lung compared with those following free‐EDV treatment. It was supposed that according to our previous study [24], some exosomes like EXO and macrophage derived exosomes could be accumulated in some specific organs and significantly changed the biodistribution of its delivered drugs. EXO + EDV enhanced EDV delivery in heart and lung may serve as a potential targeted carrier and provide a good treatment for other organs diseases.

Finally, histological differences were investigated to evaluate systemic toxicity of EDV and EXO + EDV in SD rats model. As indicated in Figure 2c, compared with sham group, there was no obvious histological difference of major organs in EDV‐, EXO‐ and EXO + EDV‐treated groups, which suggested that no significant toxicity was induced by EDV, EXO and EXO + EDV. These findings also suggested that EXO + EDV was a safe carrier for ischemic stroke therapy.

### 3.3 Edaravone loaded exosomes reduced the hemolysis in vitro

**FIGURE 3:**
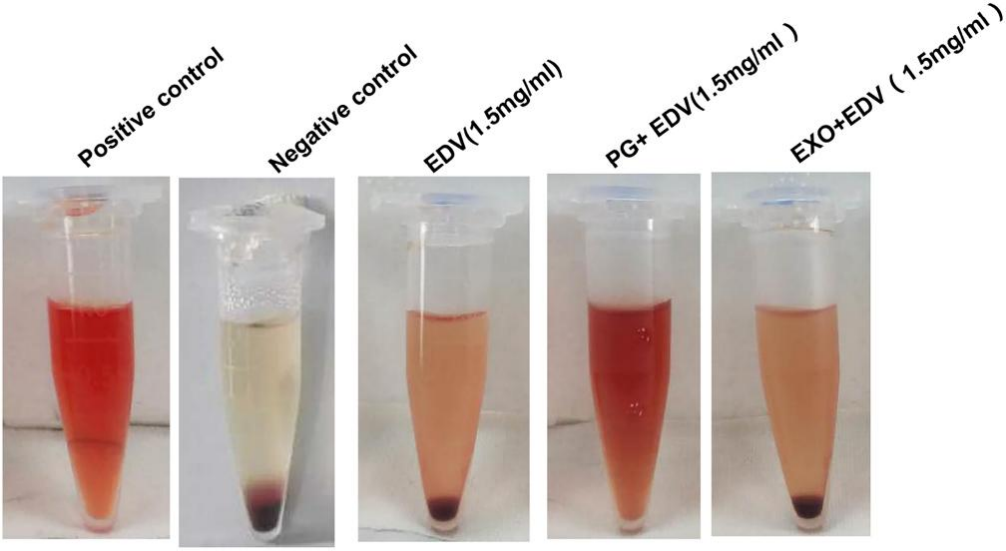
Hemolysis determined by observing the colour change of the serum and plasma after 3‐h incubation with EDV, EXO + EDV and PG + EDV, respectively

As EDV injection contained some organic solvent and often induced the strong hemolysis effects, we necessarily performed hemolysis analysis in vitro and compared the hemolysis effects between EDV + PG and EXO + EDV. The results in Figure 3 showed that when sample was treated with EDV + PG, the red blood cells were hemolyzed, and the released hemoglobin caused the serum or plasma to appear cherry red in colour. In contrat, the hemolysis effect induced by EXO + EDV was very low and the serum or plasma appeared transparent and colourless. It indicated that the hemolysis effect induced by EXO + EDV was very low, and EXO + EDV exhibited good biocompatibility and safety at the test dose under our experimental conditions.

### 3.4 Edaravone loaded exosomes enhanced uptake of edaravone via transferrin–transferrin receptor interaction

**FIGURE 4:**
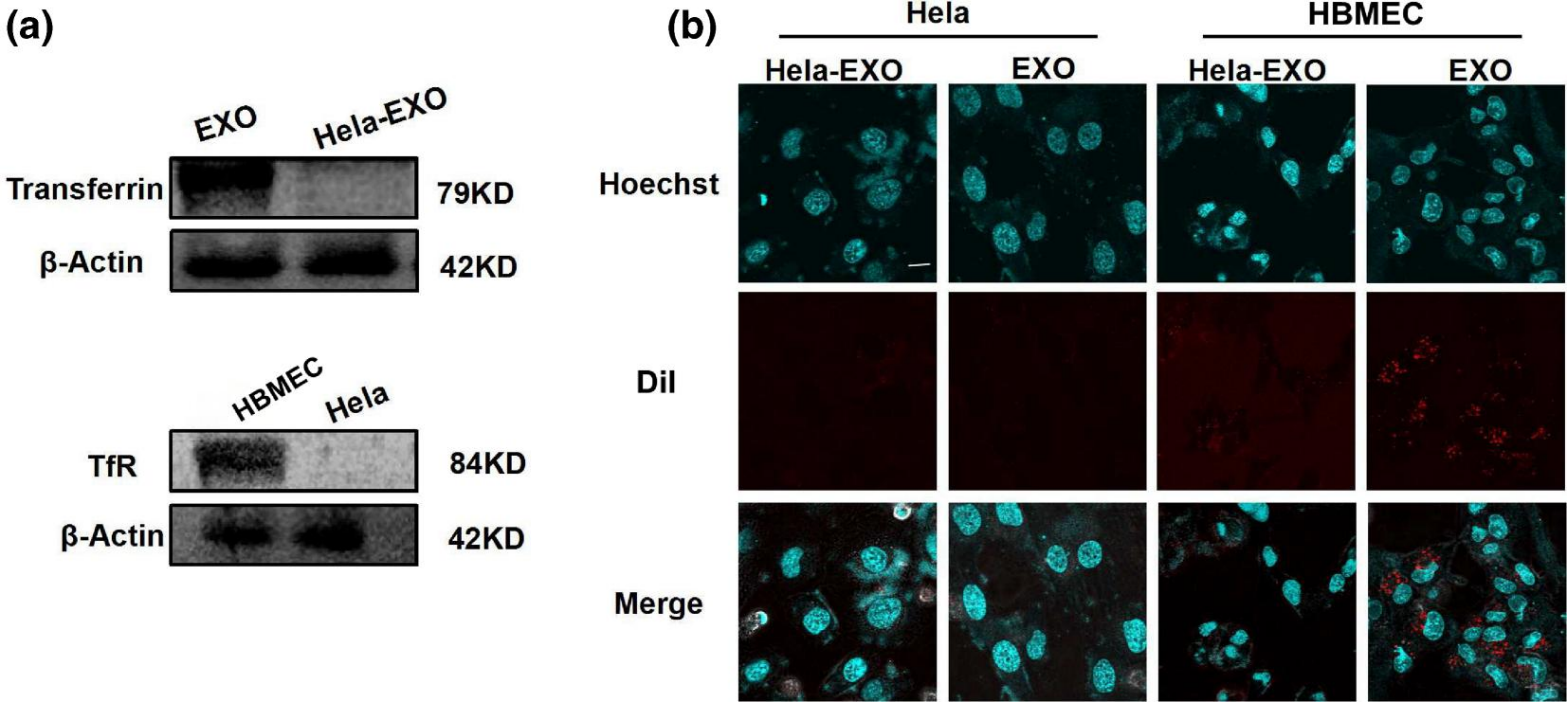
Cellular uptake of EXO and competition assay. (a) Expression of transferrin on EXO and Hela‐EXO by western blot. Expression of TfR on HBMECs and Hela cells by Western blot. (b) Confocal images of HBMECs and Hela cells after incubating with EXO and Hela‐EXO for 6 h. Nucleus was stained with hoechst (blue) for 15 min at 37°C, EXO and Hela‐EXO were labeled by DiI (red). The scale bar is 50 μm and applies to all figure parts

In order to confirm whether EXO + EDV enhanced penetration of EDV across the BBB and accelerated its accumulation in brain, the internalization process of EXO + EDV was clarified and made sure whether the transferrin on EXO had played a major role in mediating the BBB‐crossing transcytosis. The cellular uptake of DiI‐labeled EXO in the brain microvascular endothelial cell line (HBMECs, TfR positive cells) and Hela cells (TfR negative cells) was performed by confocal laser scanning microscopy, as shown in Figure 4b. The expression of TfR was evaluated on HBMECs and Hela cells (Figure 4a) by Western blot. It demonstrated that TfR was expressed higher on HBMECs than Hela cells, consistent with previous results. As shown in Figure 4b, when both cells were treated with DiI‐labeled EXO, there were increased red fluorescent spots observed in HBMECs with high expression of TfR, as compared to Hela cells without the expression of TfR. We also compared uptake capacity of transferrin positive EXO and transferrin negative Hela‐derived exosomes (Hela‐EXO) in HBMECs. It demonstrated that transferrin was expressed higher on EXO than Hela‐EXO (Figure 4a). As compared to transferrin negative Hela‐EXO, transferrin positive EXO showed the higher uptake ability in HBMECs with high expression of TfR, indicating that transferrin–TfR interaction mediated the internalization of EXO. All results clearly demonstrated that EXO could effectively promote drug accumulation in HBMECs based on the transferrin–TfR interaction, thus resulting in the enhanced intracephalic uptake and guaranteeing its neuroprotection effect.

### 3.5 Plasma exosomes were well co‐located with neurons and vascular endothelial cells in the ischemic region

**FIGURE 5:**
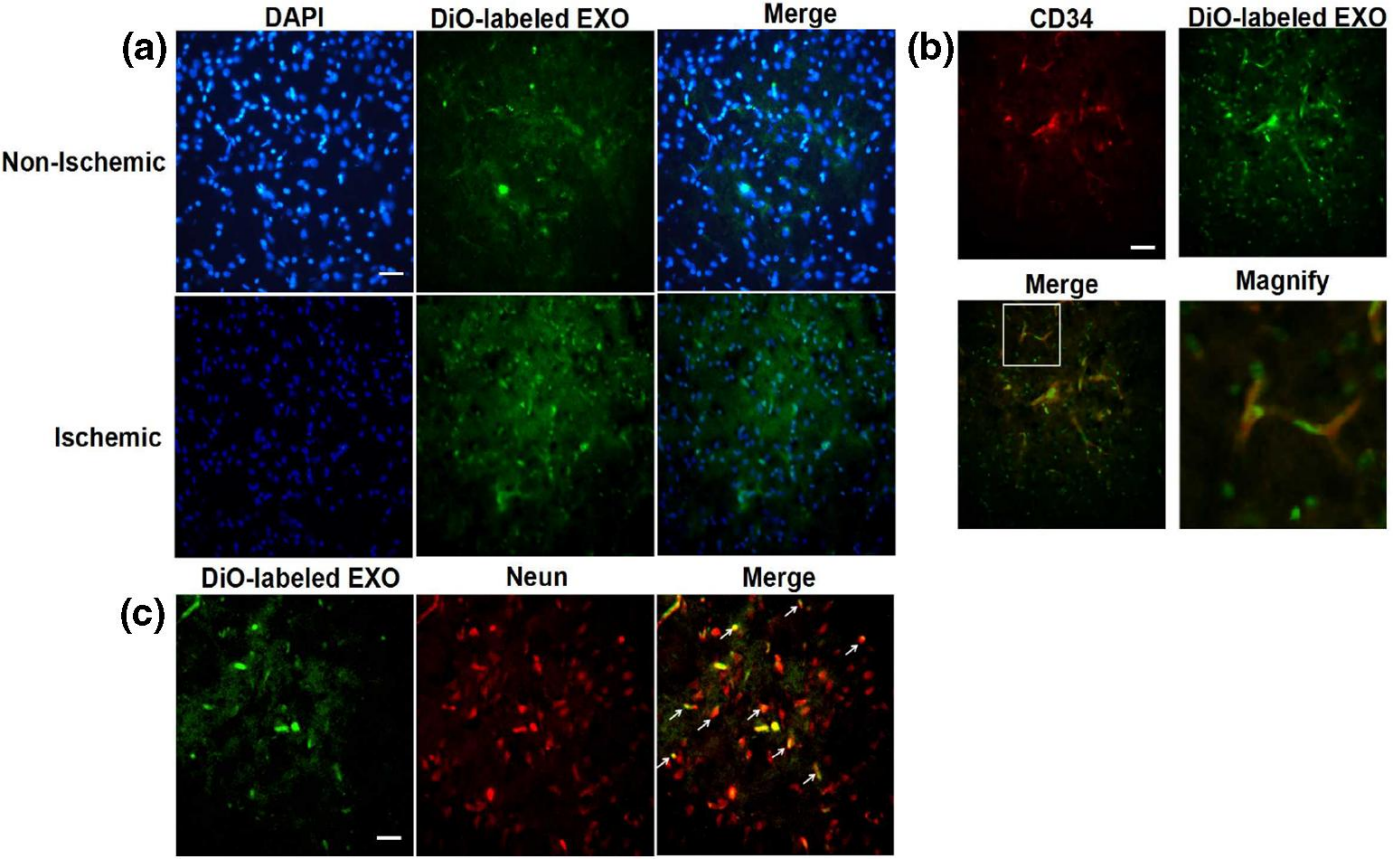
The accumulation of EXO in the ischemic region in PMCAO rats model. (a) Representative fluorescence images of ischemic brains which received the intravenous administration of DiO‐labeled EXO. Brains were dissected at 6 h after administration. The scale bar is 50 μm and applies to all figure parts. (b) Co‐location of DiO‐labeled EXO (green) with vascular endothelial cells in ischemic brain tissue. Vascular endothelial cells were stained with anti‐CD34 (red). The scale bar is 50 μm and applies to all figure parts. (c) Co‐location of DiO‐labeled EXO (green) with neurons in ischemic brain tissue. Neuron cells were stained with anti‐NeuN (red). The scale bar is 50 μm and applies to all figure parts

In order to detect the brain targeting of EXO in the ischemic area. SD rats subjected to PMCAO were injected with DiO‐labeled EXO via the tail vein and the distribution of EXO in brain tissue was observed under fluorescence microscope. We found that more DiO‐labeled EXO was accumulated in the ischemic side as compared to their distribution in the non‐ischemic side (Figure 5a), indicating that EXO could be targeted to the ischemic brain tissue owing to its specific binding with HBMEC cells and increased permeability in damaged BBB. The intracellular distribution of EXO in ischemic region was further detected from the model of PMCAO. The results (Figure 5b) demonstrated that DiO‐labeled EXO was well localized with endothelial cells stained by CD34, further confirming that EXO could be selectively targeted to endothelial cells which were important components of the BBB and indicated the possibility of crossing BBB. In the meanwhile, the increased green fluorescence denoting DiO‐labeled EXO was also well merged with the red fluorescence signifying NeuN‐positive neurons (Figure 5c). It was suggested that EXO + EDV had been internalized into neurons in the brain from PMCAO and possibly exerted their impact on regulating ischemic damage.

### 3.6 Treatment of ischemic injury with exosomal neuroprotective edaravone

**FIGURE 6:**
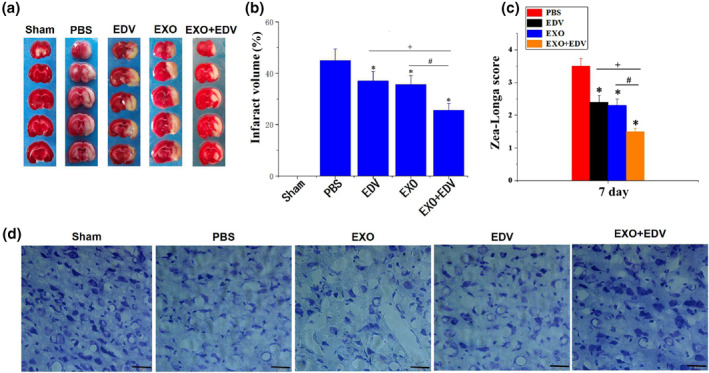
Neuroprotective role of EXO, EDV and EXO + EDV at 7 days on PMCAO rats. (a) EXO + EDV treatment reduced infarct volume. Representative brain slices with infarcts stained by 2,3,5‐triphenyltetrazolium chloride (TTC) from each group at 7 days on PMCAO rats. (b) Infarct volume on PMCAO model treated with EXO, EDV and EXO + EDV at 7 days on PMCAO rats. Data are expressed as means ± SD (*n* = 3). *P < 0.05, comparing infarct volume on PMCAO model treated with PBS. ^+^ *P* < 0.05, comparing infarct volume on PMCAO model treated with EDV with that treated with EXO + EDV. ^#^ *P* < 0.05. (c) Zea‐Longa neurological scores. Data are expressed as means ± SD (*n* = 3). *P < 0.05, comparing score on PMCAO model treated with PBS. ^+^ *P* < 0.05, comparing score on PMCAO model treated with EDV with that treated with EXO + EDV. ^#^ *P* < 0.05. (d) Representative Nissl staining of ischemic brain tissue treated with EXO, EDV and EXO + EDV at 7 days on PMCAO rats. The scale bar is 50 μm and applies to all figure parts

Since EXO significantly enhanced the bioavailability of EDV and increased its accumulated amount in the ischemic region, we next evaluated the therapeutic effects of EXO + EDV in PMCAO rats. The results demonstrated that PMCAO rats treated with EXO + EDV showed a smallest cerebral infarction volume (Figure 6a) and presented lowest neurological scores (Figure 6b), indicating that administration of EXO + EDV remarkably improved ischemic recovery of the rats in PMCAO model. Meanwhile, as compared with EDV, EXO + EDV also showed the better neuroprotective effects by inducing smaller cerebral infarction volume (37.0% vs. 25.6%, *P* < 0.05) and lower neurological scores (*P* < 0.05). Moreover, we also found that naïve EXO induced lower infarction percentage and neurological scores, but not as lower as EXO + EDV, indicating that naïve EXO exhibited a certain minor neuroprotective effects.

In order to study the protective effect of EDV on neurons in the ischemic region, the Nissl staining was used to access the number and the appearance of neurons. Nissl staining (Figure 6d) indicated that in the PBS treated PMCAO group, the pyramidal cells were sparsely distributed, the cell morphology was no longer intact and the outline was ambiguous. The number of neurons was significantly reduced as compared with the sham group. After treated with EXO, EDV and EXO + EDV respectively, the number of normal neurons was slightly increased. Moreover, EXO + EDV induced the highest number of neurons, as well as evident improvement in the integrity of basic neuronal structure. Taken together, EXO + EDV showed greater protective effects as compared to EDV treatment alone, and naïve EXO administration still played a synergistic role with EDV on ameliorating ischemic brain damage.

## 4 DISCUSSION

Ischemic stroke as the leading cause of death and disability possesses the short time window for thrombolysis (4.5 h) [25,26]. Therefore, most patients easily miss the time of thrombolytic therapy, and medication is usually used for patients who missed the time window of thrombolysis. Although some drugs against ischemic stroke show the better therapeutic effects in transient ischemia–reperfusion model, they fail to contributing more to 80% of the patients without treatment of thrombolysis in clinical trial. In fact, it is found that transient ischemia–reperfusion model can not better simulate clinical patients of ischemic stroke. Compared with the easy penetration and accumulation of drugs in transient ischemia–reperfusion model, these drugs are difficult to cross BBB and reach the ischemic brain area in clinical application [27,28]. As the pathophysiology of PMCAO induced stroke closely matches clinical symptoms in stroke patients, we use the permanent middle cerebral artery occulsion model (PMCAO) to simulate patients who are clinically failed in thrombolytic therapy. The middle cerebral artery remains obstructed throughout the treatment period, and the neuroprotective effects of EXO + EDV is studied in the state of total obstruction of the middle artery.

Edaravone as a free radical scavenger has been shown to prevent cerebral ischemic injury. In clinical trial, it is used as a potent hydroxyl radical scavenger to eliminate hydrogen oxide radicals that induce lipid peroxidation via intravenous medication. A major constraint to the yield of the therapeutic usefulness of EDV is attributed to uncertain safety, low bioavailability and BBB crossing difficulty.

In our previous study, we had fabricated EDV‐primed exosomes secreted by EDV treated mouse macrophage cells to investigate its alleviation on cerebral ischemia‐reperfusion injury [29]. Although edaravone‐loaded macrophage derived exosomes could enhance neuroprotection, the total yield of exosomes secreted by cells was very limited and the whole cell harvesting process was rather time‐consuming and expensive. In addition, the loading process of EDV was very complex; EDV had to preincubate with cells, which demanded conditional stability and sterility. Unlike few production of cell derived exosomes with longer time span, a large amount of plasma exosomes (EXO) could be easily produced in a short time by ultracentrifugation of a plenty supply of blood. Here we fabricated EXO and EDV was incorporated in EXO by simple sonification instead of coincubating with cells. We explored the potential of EDV + EXO on changing EDV pharmacokinetic profiles and enhancing its accumulation and retention time in vivo. The results demonstrated that from a safety point of view, EXO + EDV did not cause hemolysis, nor did it cause pathological changes in major organ tissues. Therefore, EXO were a promising safe drug carrier. Through sonification, more EDV was encapsulated in EXO and resulted in EE% at 55.7% and LR% at 22.0%. Therefore, relative higher drug encapsulation and loading rates facilitated the administration of EDV using EXO in vivo, thus displaying the better pharmacokinetic profile with a higher maximum plasma concentration (*C*_max_) of EDV, a substantial increase in plasma AUC with an extended half‐life (*t*_1/2_) and a significant decrease in the total CL of EDV. Furthermore, owing to the higher bioavailability, EXO loaded with a large amount of EDV resulted in a significant ischemic recovery in PMCAO model rats by inducing smaller cerebral infarction volume, presenting lower neurological scores and reducing neuronal death as compared to free EDV.

We used the high expressed transferrin on the surface of EXO to bind to the TfR on the surface of brain endothelial cells to promote BBB penetration and accelerate the targeted delivery of EDV in the ischemic brain.

Although our studies have shown EXO + EDV to be beneficial for reducing ischemic damage in animal models of PMCAO in the acute stage of stroke (first 7 days), its longer‐term sustained effects on recovery of function following stroke is still unclear. In the future, we will evaluate EXO + EDV's neuroprotective effects up to a longer period time after permanent middle cerebral artery occlusion.

## 5 CONCLUSION

We have developed a nano‐sized EDV loaded plasma exosome as a novel brain targeted drug delivery carrier. A large amount of EXO was easily isolated and collected from plasma. EDV can be effectively loaded into EXO by simple sonification and effectively specifically delivered into the ischemic brain based on transferrin–TfR interaction, thus exerting a significant neuroprotective effects in PMCAO models. These findings indicated that applications of EXO could be a hopeful approach to equip EDV and improve its neuroprotection for ischemic stroke therapy.

## CONFLICT OF INTERESTS

The authors declare no competing financial interests and all authors have approved the final article.
